# Mannitol and hyponatremia regulate cardiac ventricular conduction in the context of sodium channel loss of function

**DOI:** 10.1152/ajpheart.00211.2023

**Published:** 2024-01-12

**Authors:** Grace A. Blair, Xiaobo Wu, Chandra Bain, Mark Warren, Gregory S. Hoeker, Steven Poelzing

**Affiliations:** ^1^Graduate Program in Translational Biology, Medicine, and Health, Virginia Polytechnic Institute and State University, Roanoke, Virginia, United States; ^2^Center for Vascular and Heart Research, Fralin Biomedical Research Institute at Virginia Tech Carilion, Roanoke, Virginia, United States; ^3^Department of Biomedical Engineering and Mechanics, Virginia Polytechnic Institute and State University, Blacksburg, Virginia, United States

**Keywords:** basic science research, congenital heart disease, electrophysiology, translational studies

## Abstract

Scn5a heterozygous null (*Scn5a*^+/−^) mice have historically been used to investigate arrhythmogenic mechanisms of diseases such as Brugada syndrome (BrS) and Lev’s disease. Previously, we demonstrated that reducing ephaptic coupling (EpC) in ex vivo hearts exacerbates pharmacological voltage-gated sodium channel (Na_v_)1.5 loss of function (LOF). Whether this effect is consistent in a genetic Na_v_1.5 LOF model is yet to be determined. We hypothesized that loss of EpC would result in greater reduction in conduction velocity (CV) for the *Scn5a*^+/−^ mouse relative to wild type (WT). In vivo ECGs and ex vivo optical maps were recorded from Langendorff-perfused *Scn5a*^+/−^ and WT mouse hearts. EpC was reduced with perfusion of a hyponatremic solution, the clinically relevant osmotic agent mannitol, or a combination of the two. Neither in vivo QRS duration nor ex vivo CV during normonatremia was significantly different between the two genotypes. In agreement with our hypothesis, we found that hyponatremia severely slowed CV and disrupted conduction for 4/5 *Scn5a*^+/−^ mice, but 0/6 WT mice. In addition, treatment with mannitol slowed CV to a greater extent in *Scn5a*^+/−^ relative to WT hearts. Unexpectedly, treatment with mannitol during hyponatremia did not further slow CV in either genotype, but resolved the disrupted conduction observed in *Scn5a*^+/−^ hearts. Similar results in guinea pig hearts suggest the effects of mannitol and hyponatremia are not species specific. In conclusion, loss of EpC through either hyponatremia or mannitol alone results in slowed or disrupted conduction in a genetic model of Na_v_1.5 LOF. However, the combination of these interventions attenuates conduction slowing.

**NEW & NOTEWORTHY** Cardiac sodium channel loss of function (LOF) diseases such as Brugada syndrome (BrS) are often concealed. We optically mapped mouse hearts with reduced sodium channel expression (*Scn5a*^+/−^) to evaluate whether reduced ephaptic coupling (EpC) can unmask conduction deficits. Data suggest that conduction deficits in the *Scn5a*^+/−^ mouse may be unmasked by treatment with hyponatremia and perinexal widening via mannitol. These data support further investigation of hyponatremia and mannitol as novel diagnostics for sodium channel loss of function diseases.

## INTRODUCTION

Since the early 2000s, murine models with loss of function (LOF) of the cardiac isoform of the voltage-gated sodium channel (Na_v_1.5) have been studied in hopes of elucidating the mechanisms of arrhythmogenesis in diseases such as Brugada syndrome (BrS) and Lenegre disease (Lev’s disease; 1–5). However, previous reports have not reached a consensus as to whether a mouse with 50% reduced Na_v_1.5 function has slower ventricular conduction velocity (CV) than wild-type (WT) animals under normonatremic conditions. Initial characterizations presented no significant difference in CV relative to the WT mouse (2, 3), whereas subsequent studies report reduced CV in the heterozygous animal relative to WT (4, 5). This discrepancy highlights the importance of experimental conditions in making such conduction measurements, and also parallels clinical evidence of concealed phenotypes in Na_v_1.5 LOF diseases such as BrS (6).

Our laboratory has previously shown that changes in the osmotic or electrolyte composition of Langendorff perfusion solutions can modulate CV slowing in multiple ex vivo models of impaired ventricular conduction. Using flecainide-treated guinea pig hearts as a pharmacological model of Na_v_1.5 LOF, we demonstrated that the osmotic agent mannitol could exacerbate CV slowing by inducing intercalated disc dehiscence (7). In another study, increasing perfusate potassium concentration (indirectly inducing Na_v_1.5 LOF through increased resting membrane potential and sodium channel inactivation) and reducing sodium concentration preferentially slowed CV in ex vivo connexin-43 (Cx43) heterozygous mouse hearts relative to WT (8). Both of these previous findings support a mechanism of CV modulation via altered ephaptic coupling (EpC). The theory of EpC posits that the activation of a sodium current on one side of a small intercellular cleft [such as the intercalated disc nanodomain known as the perinexus (9)] results in a more negative extracellular cleft potential, transactivating the distal sodium channels and propagating conduction of the action potential. Accordingly, widening junctional extracellular spaces can reduce this mode of sodium channel transactivation and decrease CV. In addition, reducing extracellular sodium can theoretically reduce CV via EpC by reducing the driving force for sodium.

Interestingly, numerous clinical case reports describe that a BrS ECG phenotype can be unmasked by hyponatremia (10–14). Although many of these studies report the phenomenon as a BrS “phenocopy” because of its transient nature (resolving upon recovery of serum sodium concentration), they nevertheless highlight the efficacy of hyponatremia as a substrate for electrophysiological conduction disturbance. Therefore, patients with Na_v_1.5 LOF may be more vulnerable to changes in serum sodium concentration relative to unaffected individuals.

Considering our previous publications, as well as clinical case reports of patients with BrS, we hypothesize that the heart’s extracellular osmotic and ionic composition plays a pivotal role in modulating conduction, particularly in the context of Na_v_1.5 LOF. This study was conducted to assess whether mice heterozygous null for the *Scn5a* gene encoding Na_v_1.5 (*Scn5a*^+/−^) are more susceptible to CV slowing than WT in response to mannitol-induced perinexal widening and decreased extracellular sodium. We found that conduction is not significantly different between *Scn5a*^+/−^ and WT mouse hearts during normonatremia, but *Scn5a*^+/−^ hearts do demonstrate greater slowing after mannitol treatment and unidirectional conduction block during hyponatremia. Surprisingly, we found that mannitol attenuated conduction slowing during hyponatremia for both genotypes.

## METHODS

### Mouse Model

Original breeder wild-type (WT) and *Scn5a*^+/−^ mice on a 129/SvEv-C57BL/6 background were acquired from Taconic biosciences and bred onsite in Roanoke, VA. Mice of both sexes, age 14–30 wk, were used in all protocols, though due to availability at the time of experimentation, the distribution of sexes was uneven within treatment groups. All protocols were approved by the Institutional Animal Care and Use Committee at Virginia Polytechnic Institute and State University and conform to the guidelines of the National Institutes of Health’s Guide for the Care and Usage of Laboratory Animals.

### RNA Purification and qRT-PCR

RNA was isolated from snap-frozen left ventricles. Tissues were lysed in TRIzol (Thermo Fisher, Cat. No. 15596026) per manufacturer’s protocol and separated into phases using Phase lock gel tubes (Fisher, Cat. No. NC1093153). The top clear aqueous layer was further purified using the NucleoSpin RNA kit (MACHEREY-NAGEL, Cat. No. 740955), and cDNA was generated using the SuperScript IV First-Strand Synthesis System (Thermo Fisher, Cat. No. 18091050), according to the manufacturers’ instructions. cDNA was used at a concentration of 20 ng per qRT-PCR reaction with gene-specific primers (SCN5A forward: 5′-GCT GCC AGA TCT CTA TGG CAA CCC-3′, SCN5A reverse: 5′-CAA GGC ATT GGT GGC ACT GAA CC-3′; HPRT1 forward: 5′-CCC CAA AAT GGT TAA GGT TGC, HPRT1 reverse: 5′-AAC AAA GTC TGG CCT GTA TCC-3′; GAPDH forward: 5′-AAT GGT GAA GGT CGG TGT G-3′, GAPDH reverse: 5′-GTG GAG TCA TAC TGG AAC ATG TAG-3′) and SYBR Select Master Mix for CFX (Thermo Fisher, Cat. No. 4472937). All samples were normalized to the combined threshold values of HPRT1 and GAPDH together as housekeeping genes and data are represented relative to WT Scn5a expression.

### Western Blot Analysis

Left ventricular tissue samples were snap frozen and Western blotting was performed. Briefly, the samples were homogenized in RIPA lysis buffer, containing 50 mM Tris (pH 7.4), 150 mM NaCl, 1 mM EDTA, 1% Triton X-100, 1% sodium deoxycholate, 0.1% sodium dodecyl sulfate, 200 μM Na_3_VO_34_, 1 mM NaF, and 5.6 mM *N*-ethylmaleimide, supplemented with Roche Protease Inhibitor Cocktail (Millipore Sigma, Cat. No. 4693159001). Protein concentration was determined by a Bio-Rad DC protein assay (Cat. No. 500-0112) and concentrations were normalized before analysis. Electrophoresis was performed to separate proteins that were then transferred to a PVDF membrane, blocked with 5% bovine serum albumin for 1 h at room temperature, and incubated overnight with a primary antibody against either the principal ventricular gap junction protein Cx43 phosphorylated at Ser368 [pCx43, Cell Signaling Technology, Cat. No. 3511S, validated in Solan et al. (15), 1:1,000 dilution] or against the voltage-gated sodium channel protein Na_v_1.5 [Cell Signaling Technology, Cat. No. 14421s, validated in Saadeh et al. (16), 1:1,000 dilution], at 4°C. The membranes were then washed and incubated with secondary antibody (Abcam, Cat. No. ab6721, 1:10,000 dilution) at room temperature for 1 h. After washing, bound antibody was detected using West Pico Plus chemiluminescent substrate (Thermo Fisher, Cat. No. 34579) and imaged using the Li-Cor Odyssey Fc system. Membranes were stripped with ReBlot Plus (Millipore Sigma, Cat. No. 2504) according to manufacturer’s instructions, blocked in Intercept Blocking Buffer (Li-Cor, Cat. No. 927-60001) at room temperature for 1 h and incubated with primary antibodies against total Cx43 [Millipore Sigma, Cat. No. C6219, validated in George et al. (17), 1:5,000 dilution] and GAPDH (VWR, Cat. No. 101983-284, 1:5,000 dilution). Membranes were then washed and incubated with secondary antibodies for 1 h (Li-Cor, Cat. No. 925-32211, 1:10,000 dilution; and Li-Cor, Cat. No. 925-68070, 1:10,000 dilution) and washed again. Membranes were again imaged using the Li-Cor Odyssey Fc system to determine protein expression. Total Cx43 and Na_v_1.5 protein expression were normalized to GAPDH and pCx43 was normalized to total Cx43.

### In Vivo ECG

Noninvasive, unanesthetized ECG recordings were acquired using an ECGenie system (Mouse Specifics, Framingham, MA). Mice of each genotype (WT, *n* = 5 males, 1 female, *Scn5a*^+/−^ *n* = 5 males, 1 female) were placed on the recording platform for a habituation period of 15 min before recording commenced. Recording continued until 10 s of stable ECG signal was acquired. QRS duration was calculated from the average of all ECG traces recorded over the 10-s acquisition. Heart rate averaged 724 beats/min. Signals were acquired using EzCG software (Mouse Specifics, Framingham, MA), and signal processing was performed using LabChart ECG Analysis (ADInstruments, Colorado Springs, CO).

### Langendorff-Perfused Heart Preparations

#### Mouse.

Mice (*n* = 5 males, 5 females, *Scn5a*^+/−^, *n* = 6 males, 6 females WT) were anesthetized by 4% isoflurane in O_2_; anesthesia was confirmed by the absence of response to toe pinch. Cervical dislocation was then performed and immediately followed by thoracotomy and excision of the heart. The ex vivo heart was retrogradely perfused via the aorta using a rolling pump at 1.5 mL/min. The perfusate consisted of oxygenated solution, containing (in mM) 1.8 CaCl_2_·2H_2_O, 144.5 NaCl, 1.0 MgCl_2_·6H_2_O, 5.5 NaOH, 1.2 NaH_2_PO_4_, 4.0 KCl, 5.5 dextrose, and 10 HEPES (titrated to pH 7.4). Total sodium content of this solution was 151.7 mM Na^+^, which will be referred to as murine “normonatremia,” or nominally referred to as “152 mM Na^+^.” After cannulation, hearts were immersed in perfusion solution within a custom-designed optical mapping bath maintained at 37°C (18).

#### Guinea pig.

Adult male Hartley albino guinea pigs (Hilltop, Scottdale, PA; male; age, 14–16 mo old; *n* = 6) were anesthetized using 4% isoflurane in O_2_. After loss of response to toe pinch, thoracotomy was performed, and the heart was rapidly excised and cannulated (<4 min). The ex vivo heart was retrogradely perfused via the aorta with a crystalloid solution, consisting of (in mM) 140 NaCl, 5.0 NaOH, 4.56 KCl, 1.25 CaCl_2_·2H_2_O, 5.5 dextrose, 0.7 MgCl_2_·6H_2_O, and 10 HEPES. Total sodium content of this solution was 145 mM Na^+^, which will be referred to as guinea pig “normonatremia.” The perfusate was equilibrated to a pH of 7.4 using NaOH or HCl, as necessary, and was oxygenated and maintained at 37°C. Perfusion occurred at 20 mL/min. After cannulation, atria were removed to prevent competitive stimulation with the pacing electrode, and hearts were immersed in perfusion solution within a custom-designed optical mapping bath, also maintained at 37°C (18).

### Optical Mapping

For both species, conduction velocity (CV) was quantified by optical mapping using the voltage sensitive dye, di-4-ANEPPS (7.5 μM, Biotium, Cat. No. 61010). A custom Matlab program was used to quantify transverse and longitudinal CV (CVT and CVL, respectively) in a single action potential from each optical map, as previously described (19). Severe conduction slowing was a priori defined as CV less than 0.05 m/s based on previous optical mapping experiments defining 0.05 m/s as conduction block (20) or extremely slow conduction in ischemic border zone tissue (21).

#### Mouse.

After a 10-min stabilization period following cannulation, the preparation was stained with di-4-ANEPPS by retrograde aortic perfusion for ∼12 min. The electromechanical uncoupler blebbistatin (10 µM, Sigma, B0560) was added to all solutions following dye loading. A 20-min dye washout period was provided using baseline solution to remove excess dye. For each image acquisition, the tissue was excited by 510 nm light from an LED (LEX2-LZ4, SciMedia), and the emitted light was transmitted through a 610 nm long-pass filter (Andover Corp.). Filtered emissions were captured by a Micam Ultima L-type CMOS camera over 100 × 100 pixels and a magnification of ×1.60, with a field of view of 6.25 mm × 6.25 mm. Hearts were stimulated using a unipolar Ag-Ag/Cl electrode on the anterior epicardium, with the ground lead placed in the rear of the bath. Pacing was performed at a basic cycle length of 150 ms with a 1-ms cathodal pulse at 1.5× the minimum current required to elicit tissue excitation. In one cohort, baseline solution was perfused for 20 min during dye washout, followed by the same solution with the addition of mannitol (Sigma-Aldrich M4125, d-mannitol, 26.1 g/L) for 30 min. In the second cohort, baseline solution was perfused for 20 min during dye washout, followed by 30 min of perfusion with a hyponatremic version of baseline solution (120 mM Na^+^), followed by 30 min of perfusion with the addition of mannitol (26.1 g/L) to the hyponatremic solution. An additional cohort of *n* = 4 male WT mice were subjected to a time control perfusion protocol in which they were perfused with baseline solution for 60 min after dye washout and optically mapped at 10-min intervals. Optical maps were recorded at a 1 kHz sample rate for a duration of 2 s during both intrinsic activity and steady-state pacing (150-ms basic cycle length) after each perfusion solution. All representative isochrone maps presented in results illustrate activation time in 2-ms time steps. Isochrone maps include conduction vectors ranging from 0.01 to 1.0 m/s. Activation time maps containing conduction vectors ≤ 0.05 m/s were categorized as having severe conduction delay, as depicted in Fig. 4*A* (representative *Scn5a*^+/−^ heart treated with 120 mM Na^+^).

#### Guinea pig.

After a 10-min stabilization period following cannulation, the preparation was stained with di-4-ANEPPS by retrograde aortic perfusion for ∼5 min. The electromechanical uncoupler blebbistatin (10 µM, Sigma, B0560) was added to all solutions following dye loading. A 10-min dye washout period was provided using baseline solution to remove excess dye. For each image acquisition, the tissue was excited by 510 nm light from an LED (LEX2-LZ4, SciMedia), and the emitted light was transmitted through a 610 nm filter. Filtered emissions were captured by a Micam Ultima L-type CMOS camera over 100 × 100 pixels and a magnification of ×0.63, with a field of view of 15.9 mm × 15.9 mm. Hearts were stimulated using a unipolar Ag-Ag/Cl electrode on the anterior epicardium, with the ground lead placed in the rear of the bath. Pacing was performed at a basic cycle length of 300 ms with a 5 ms wide cathodal pulse at 1.5× the minimum current required to elicit tissue excitation. After dye washout, all hearts were perfused with baseline solution for 10 min, followed by 15 min of perfusion with a hyponatremic version of “baseline” (120 mM Na^+^) and the sodium channel inhibitor flecainide (0.5 µM). Finally, mannitol (26.1 g/L) was added to the hyponatremia and flecainide solution, and this was perfused for a further 15 min. Optical maps were recorded at a 1-kHz sampling rate for a duration of 2 s during both intrinsic activity and steady-state pacing at 300-ms basic cycle length after each perfusion solution. All representative isochrone maps presented in results illustrate activation time in 3-ms time steps.

### Transmission Electron Microscopy

Anterior epicardial tissue from the base of the left ventricle was collected from hearts after conclusion of the optical mapping experiments. Tissue was cut into 1-mm^3^ cubes which were fixed in 2.5% glutaraldehyde at 4°C for 24 h, and then washed thrice and stored in phosphate-buffered saline (PBS) at 4°C. As previously described (8), samples were prepared for transmission electron microscopy (TEM) and imaged using a JEM JEOL 1400 Electron Microscope at ×150,000 magnification. To quantify perinexal width (*W*_P_), at least 15 images were collected and analyzed per heart using a previously described custom Matlab program (22). The average perinexal width measured between 30 and 105 nm from the edge of the gap junction plaque is reported as *W*_P_.

### Statistical Analysis

All statistical analysis was performed using Graphpad Prism 8 (San Diego, CA). Specific *n* values and details of specific statistical tests used for each experiment are included in figure legends. Normality of each group was assessed using the Shapiro–Wilk test. Groups that did not pass the normality test were further tested for the probability of being sampled from a Gaussian versus lognormal distribution (via the ’Compare normal and lognormal’ assessment in Graphpad Prism) and this result determined whether the data would be transformed to a lognormal distribution or used as if normal. This was followed by the paired two-tailed Student’s *t* test. For unpaired data, an unpaired *t* test was used when variances were equal, and Welch’s correction was used when variances were unequal. For the hyponatremia and combined hyponatremia/mannitol interventions, distributions were normal, and repeated-measures one-way ANOVAs were performed with the Geisser–Greenhouse correction (thereby not assuming sphericity), followed by the Tukey’s multiple comparisons test, with individual variances computed for each comparison. All summary data are reported as means ± SD. All tests were considered statistically significant if *P* < 0.05.

## RESULTS

### Baseline Characteristics

**Figure 1.**
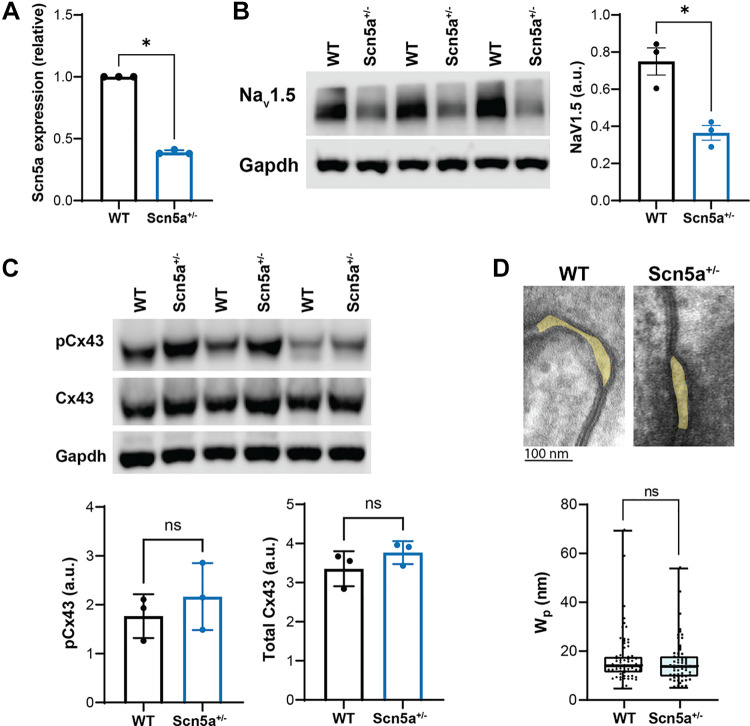
Characterizing the *Scn5a*^+/−^ mouse heart. *A* and *B*: relative to wild type (WT), the *Scn5a*^+/−^ mouse expresses ∼60% reduced mRNA and ∼52% reduced voltage-gated sodium channel (Na_v_)1.5 protein. Na_v_1.5 protein expression is normalized to GAPDH. *n* = 2 males, 1 female WT; *n* = 2 males, 1 female *Scn5a*^+/−^ hearts, significance determined by Student’s *t* test, *P < 0.05 compared with WT. *C*: no significant difference was measured in total connexin-43 (Cx43) expression (normalized to GAPDH) or Cx43 phosphorylation at Ser368 (normalized to total Cx43) between the genotypes. *n* = 2 males, 1 female WT; *n* = 2 males, 1 female *Scn5a*^+/−^ hearts. *P* = not significant (n.s.) via one-way ANOVA. *D*: perinexal width, highlighted in yellow, was not significantly different between WT and *Scn5a*^+/−^ hearts under baseline conditions. *n* = 2 males, 1 female WT; *n* = 2 males, 1 female *Scn5a*^+/−^ hearts, *n* = 15 images/heart. Significance determined by nested *t* test; *P < 0.05 compared with WT.

As expected, mRNA expression of the *Scn5a* gene was reduced by ∼60% in the *Scn5a*^+/−^ mouse heart relative to WT, as measured with qRT PCR (Fig. 1*A*; full blots provided in Supplemental Fig. S1, see https://doi.org/10.6084/m9.figshare.24944835). In addition, *Scn5a*^+/−^ hearts express a 50% reduction in Na_v_1.5 relative to WT as measured by Western blot. These results are in agreement with previous literature, which additionally demonstrated a 30–50% reduction in the voltage-gated sodium current in isolated cardiomyocytes of a *Scn5a*^+/−^ mouse as described by Martin et al. (23). In addition, neither expression of the principle ventricular gap junction protein connexin 43 (Cx43) nor phosphorylated connexin 43 (pCx43) were significantly different between WT and *Scn5a*^+/−^ mouse hearts, seen in Fig. 1, *B* and *C*. It has previously been observed that the β 1 subunit of voltage-gated sodium channels may play a role in maintaining the structural integrity of the intercalated disc, particularly with the perinexal nanodomain (24). We therefore used transmission electron microscopy to evaluate perinexal width and found no significant difference between the two genotypes (Fig. 1*D*).

### In Vivo Conduction Measurements

**Figure 2.**
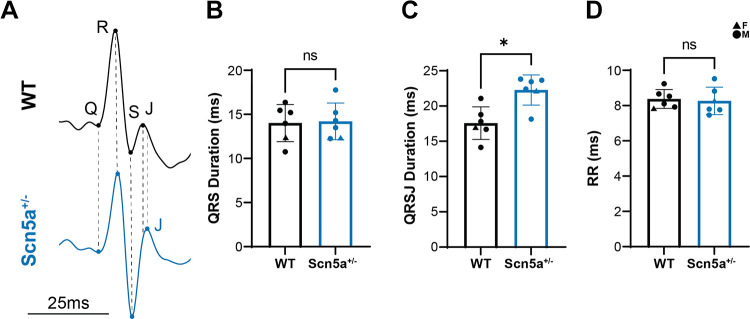
In vivo ventricular conduction is not significantly different between *Scn5a*^+/−^ and wild-type (WT) mice. *A*: typical example of an ECG trace acquired with the ECGenie system for the WT (black) and *Scn5a*^+/−^ (blue) mouse. *B*: QRS duration is not significantly different between the two genotypes, though QRSJ is significantly longer in the *Scn5a*^+/−^ mouse (*C*). *D*: RR duration is not different between the WT and *Scn5a*^+/−^ mouse. *n* = 5 males, 1 female WT; 5 males 1 female *Scn5a*^+/−^; significance was determined using Student’s *t* test, *P < 0.05 compared with WT.

An in vivo, nonanesthetized ECG study revealed no measurable difference in QRS width between the WT and *Scn5a*^+/−^ mice, suggesting little to no ventricular CV slowing in the hearts of intact *Scn5a*^+/−^ animals (Fig. 2, *A* and *B*). The QRSJ interval, however, was significantly longer in the heterozygous group, potentially implicating differences in repolarization distribution across the murine heart (25; Fig. 2*C*). No difference in RR interval was measured between the genotypes, suggesting no differences in sinoatrial function at baseline. Resolution of this technique was not fine enough to identify P waves in this model or subsequently measure PR intervals to further assess atrioventricular and specialized conduction system dysfunction.

### Normonatremia: Ex Vivo Ventricular Conduction Slowing

**Figure 3.**
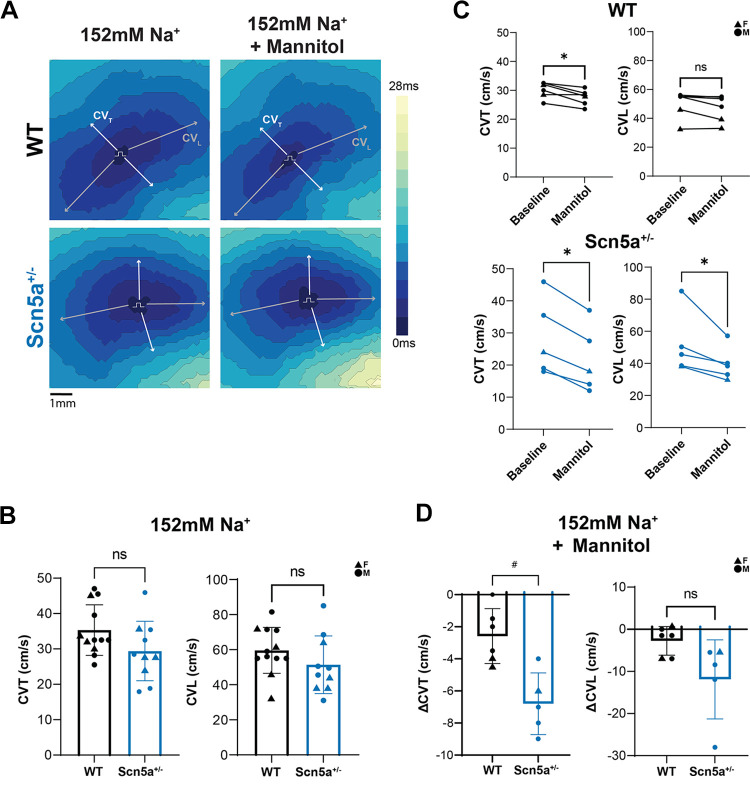
Conduction velocity (CV) of *Scn5a*^+/−^ hearts is not significantly slower than wild type (WT) at baseline, but more sensitive to CV slowing due to mannitol challenge. *A*: representative activation time maps for *Scn5a*^+/−^ and WT ventricles at normonatremia before and after mannitol challenge. Isochrone lines represent 2-ms intervals. Pulse symbol (П) indicates site of electrode placement for pacing. Arrows indicate vectors used for CV calculations. *B*: neither transverse CV (CVT) nor longitudinal CV (CVL) is significantly slower in the *Scn5a*^+/−^ heart relative to WT. *n* = 8 males, 4 females WT; 6 males, 4 females *Scn5a*^+/−^, significance determined by Welch’s *t* test, *P < 0.05 compared with WT. *C*: perfusion with mannitol significantly slowed CVT in WT and CVT and CVL in *Scn5a*^+/−^ hearts. *D*: CVT slows to a greater extent in *Scn5a*^+/−^ relative to WT hearts after mannitol challenge. *n* = 4 males, 2 females WT; 4 males, 1 female *Scn5a*^+/−^. Significance determined by Welch’s *t* test, *P < 0.05 compared with CV of WT; #*P* < 0.05 compared with ΔCV of WT.

Hearts were Langendorff perfused and optically mapped to calculate an ex vivo measure of ventricular CV. During perfusion of the baseline, normonatremic solution, CV did not significantly differ between WT and *Scn5a*^+/−^ hearts in either the transverse (CVT WT: 35.3 ± 7.1, *Scn5a*^+/−^: 29.4 ± 8.4 cm/s) or longitudinal direction (CVL WT: 59.6 ± 13.1, *Scn5a*^+/−^: 51.4 ± 16.4 cm/s). These results are depicted as representative data in Fig. 3*A*, *left*, and summary data in Fig. 3*B*.

Previous work from our laboratory identified mannitol as an osmotic agent capable of inducing edema within the intercalated disc and resulting in slowed CVT (19). Representative data (Fig. 3*A*, *right*) and summary data (Fig. 3*C*) demonstrate that mannitol significantly slows CVT of both WT and *Scn5a*^+/−^ hearts, though to a significantly greater extent in *Scn5a*^+/−^ hearts (ΔWT: −2.6 ± 1.7, Δ*Scn5a*^+/−^: −6.8 ± 1.9 cm/s relative to 152 mM Na^+^ alone). CVL decreased relative to baseline in both WT and *Scn5a*^+/−^ hearts after mannitol treatment (ΔWT: −2.8 ± 3.4, Δ*Scn5a*^+/−^: −11.9 ± 9.3 cm/s), but the difference between genotypes was not significant. Perfusion of baseline solution for 60 min resulted in no significant change in either CVT or CVL (Supplemental Fig. S2; see https://doi.org/10.6084/m9.figshare.24944835). Therefore, the data suggest that CV in *Scn5a*^+/−^ hearts, at least in the transverse direction of propagation, is more sensitive to mannitol challenge.

### Hyponatremia: Ventricular Conduction Slowing and Block

**Figure 4.**
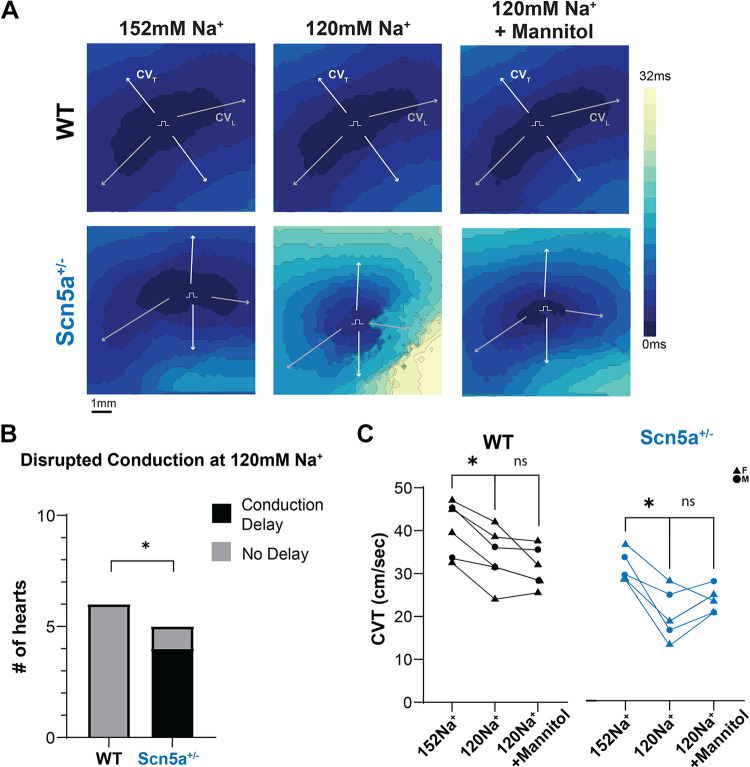
Mannitol attenuates hyponatremia-induced conduction velocity (CV) slowing and resolves conduction block in *Scn5a*^+/−^ hearts. *A*: representative activation time maps during baseline, hyponatremia, and combined hyponatremia and mannitol conditions. Isochrone lines represent 2 ms. Pulse symbol (П) indicates site of electrode placement for pacing. Arrows indicate vectors used for CV calculations. *B*: wild type (WT) does not exhibit severe conduction delay during hyponatremia, but 4/5 *Scn5a*^+/−^ exhibit severe conduction delay under these conditions. Significance determined by Fisher’s exact test; *P < 0.05 compared with WT. *C*: WT and *Scn5a*^+/−^ hearts have significantly slowed transverse CV (CVT) in response to hyponatremia, but no significant change in CVT after the addition of mannitol. *n* = 2 males, 4 females WT; 2 males, 3 females *Scn5a*^+/−^, significance determined by repeated-measures one-way ANOVA with the Geisser–Greenhouse correction (for unequal variability of differences), followed by the Tukey’s multiple comparisons test; *P < 0.05 compared with 152 mM Na^+^ condition.

Hyponatremia has been clinically identified as a potential trigger for a BrS ECG pattern (10–14). We therefore perfused WT and *Scn5a*^+/−^ hearts with a hyponatremic solution containing 120 mM Na^+^, comparable with the degree of hyponatremia recorded in case studies (10–14). Representative data in Fig. 4*A* reveal significant conduction slowing with hyponatremia relative to 152 mM Na^+^ in WT (ΔCVT: −6.6 ± 2.7, ΔCVL: −8.5 ± 9.2 cm/s) and *Scn5a*^+/−^ (ΔCVT: −10.8 ± 4.9, ΔCVL: −15.8 ± 19.0 cm/s) hearts. Interestingly, hyponatremia resulted in disrupted conduction in the left ventricle of 4/5 *Scn5a*^+/−^ hearts during epicardial pacing, as observed by the isochrone line crowding and severe conduction delay depicted in Fig. 4*A*, *bottom*, *middle*. Conversely, this hyponatremia-induced disrupted conduction presented in none of the WT hearts (0/6). The data suggest that hyponatremia slows CV and increases the likelihood for conduction failure and unidirectional block in the presence of Na_v_1.5 LOF.

### Hyponatremia and Mannitol: Attenuated Conduction Slowing and Rescue from Conduction Block

Representative optical maps of activation time (Fig. 4*A*) and summary data (Fig. 4*C*) demonstrate that WT hearts treated with hyponatremia and mannitol did not significantly slow relative to hearts perfused with a hyponatremic solution alone. These results are in contrast to CV slowing observed in response to mannitol treatment in hearts perfused with 152 mM Na^+^ (Fig. 3).

Unexpectedly, conduction block caused by hyponatremia alone resolved for all affected *Scn5a*^+/−^ hearts after treatment with a combination of hyponatremia and mannitol. This is again in contrast to results of mannitol treatment during normonatremia, which resulted in significant slowing in both CVT and CVL (Fig. 3). In conclusion, mannitol attenuates CV slowing because of hyponatremia for both *Scn5a*^+/−^ and WT hearts.

### Comparative CV Response to Hyponatremia and Mannitol in Guinea Pig

**Figure 5.**
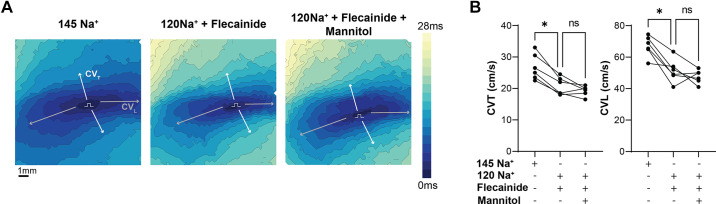
Attenuation of conduction velocity (CV) slowing during combined hyponatremia and mannitol challenge is not species specific. *A* and *B*: significant reductions in transverse CV (CVT) and longitudinal CV (CVL) are observed after perfusion with 120 mM Na^+^ and flecainide in ex vivo guinea pig hearts. Addition of mannitol does not show additive CV slowing effect in the context of hyponatremia and sodium channel inactivation. *n* = 6 males, significance determined by one-way ANOVA with Tukey’s multiple comparisons test; *P < 0.05 compared with 145 mM Na^+^ condition.

Our laboratory previously demonstrated that isolated guinea pig hearts treated with both flecainide and mannitol (maintained at a physiological sodium concentration) show an additive effect on CV slowing, similar to the findings aforementioned in mouse hearts perfused with normonatremia and then mannitol. Prior time control experiments using baseline solution show no change in CV over the same treatment duration (26). However, it is unknown if the unexpected result that mannitol attenuates CV slowing because of hyponatremia is specific to mice or can be replicated in a larger animal species. Representative images of the optical maps for guinea pig hearts perfused with normonatremia, hyponatremia and flecainide, and a hyponatremia-flecainide-mannitol solution are shown in Fig. 5*A*. Summary data (Fig. 5*B*) during hyponatremia demonstrate that the addition of mannitol to flecainide-treated guinea pig hearts at 120 mM Na^+^ does not further reduce CV. This parallels results in the *Scn5a*^+/−^ mouse model aforementioned, suggesting that mannitol-induced CV slowing is dependent on extracellular sodium concentration.

## DISCUSSION

This study was conducted to determine whether a congenital mouse model of Na_v_1.5 LOF would be more susceptible to ventricular conduction slowing in response to reduced EpC relative to WT littermates. Specifically, we measured ventricular conduction in in vivo and ex vivo WT and *Scn5a*^+/−^ mouse hearts, and challenged the ex vivo heart preparations with perinexal widening via mannitol during normo- and hyponatremia. We found that CV is not significantly different between WT and *Scn5a*^+/−^ hearts under baseline conditions in either the transverse or longitudinal direction. However, the *Scn5a*^+/−^ hearts are more susceptible to CV slowing via mannitol than WT at normonatremia, particularly in the transverse direction of conduction. This is consistent with the propagation of electrical wavefronts through more intercalated discs and therefore more ephapses per unit length in the transverse relative to longitudinal direction across the myocardium. Because conduction across the intercalated disc is thought to be slower than through the myocyte itself (27), the greater number of junctions encountered in transverse versus longitudinal propagation should multiply the effect of manipulating junctional communication on cardiac conduction. Although experimental evidence supports this hypothesis, this has not been tested computationally. *Scn5a*^+/−^ hearts also experience severe unidirectional conduction delay during hyponatremia, whereas WT experience reduced CV but not severe delay. Interestingly, although mannitol slowed CV during normonatremia in both WT and *Scn5a*^+/−^ hearts, mannitol did not further slow CV during hyponatremia. This effect was also independent of Na_v_1.5 expression levels.

Previously, other laboratories have noted downregulation of Cx43 is a comorbidity in *Scn5a*^+/−^ mice that exacerbates conduction deficits during Na_v_1.5 LOF (1, 4, 28). Our group, however, failed to find such a reduction in Cx43 or pCx43 expression via Western blot. This discrepancy may be attributed to the age of the respective cohorts, as age-associated Cx43 downregulation has been demonstrated in WT mouse cardiac tissue (29). Previous studies reporting reduced Cx43 expression used *Scn5a*^+/−^ mice > 30 wk of age, and Derangeon et al. (30) demonstrate that downregulation of Cx43 begins around the age of 45 wk. Mice in this study, however, were all between 14 and 30 wk of age. Therefore, it remains unknown whether aging itself confounds Cx43 downregulation and conduction deficits concomitant with Na_v_1.5 LOF.

Interestingly, the observation of worsening electrophysiological phenotype with increased age described in prior *Scn5a*^+/−^ mouse studies does not correspond to the clinical data for patients with BrS. To the contrary, symptomatic BrS most commonly presents in the fourth decade of life (31). Beyond this time, the prognosis for patients with BrS appears to improve with age (32–34). Therefore, even if progressive downregulation or remodeling of Cx43 secondary to Na_v_1.5 LOF is exacerbated with age, our data from this preclinical murine model suggest that other mechanisms for conduction slowing or disruption may underlie arrhythmogenesis in young adult patients with Na_v_1.5 LOF.

The osmotic agent mannitol has previously been validated as a means of widening the perinexal nanodomain and reducing ventricular CV via EpC (35, 36). We, therefore, proposed that a mannitol challenge may further exacerbate conduction slowing in the Langendorff-perfused *Scn5a*^+/−^ mouse heart. Optical mapping after administration of mannitol during normonatremia resulted in greater CV slowing in the *Scn5a*^+/−^ heart relative to WT, suggesting that the heterozygous animal is more sensitive to this form of osmotic stress. As mannitol widens *W*_P_ (19, 36, 37), but does not significantly reduce peak *I*_Na_ (38) itself, the data support the hypothesis that reduced sodium channel expression is functionally unmasked by perinexal widening via mannitol during normonatremia. Mechanistically, we propose that perinexal widening reduces ephaptic transactivation of Na_v_1.5 by attenuating extracellular potential changes, and this appears to have an additive effect with the reduced sodium current intrinsic to our mouse model.

Although it has been demonstrated that mannitol can decrease isolated cell size, which in turn could slow conduction by increasing the number of gap junctions per unit length, there exists a paradox in that mannitol also increases heart volume. Increased volume must at least be associated with an increase in cell length, and this could be expected to increase CV. Thus, the effect of mannitol on cell size and conduction remains unresolved and is an area for future investigation. Mannitol also increases bulk extracellular conductivity, but recent evidence suggests that increasing extracellular volume is not the primary mechanism by which mannitol decreases CV (39). We also recently published that mannitol significantly affected neither gap junctional coupling nor peak sodium currents, which reduces the likelihood that gap junction inhibition or alterations to sodium currents are mechanisms that well explain the results (39). Finally, mannitol inhibits the rapid component of the delayed rectifier potassium channel hERG (40, 41). We have shown that hERG inhibition can increase CV under specific conditions (42), and it is therefore possible that some of the unexpected effects with mannitol are consequent to the reduction of outward currents that oppose inward sodium.

Reducing extracellular sodium is another mechanism previously demonstrated to reduce ventricular CV in the Langendorff-perfused heart (8, 17). Interestingly, hyponatremia has also been associated with unmasking a BrS ECG pattern in human patients (10–14). We therefore tested the hypothesis that hyponatremia exacerbates the conduction phenotype of Na_v_1.5 LOF. In our study, perfusion of *Scn5a*^+/−^ hearts with a 120 mM Na^+^ solution not only exacerbated conduction slowing but also caused severely delayed conduction in the left ventricle in 4/5 heterozygous hearts. Conversely, delayed conduction was observed in 0/6 WT hearts during hyponatremia. It is also important to note that this conduction abnormality was observed under conditions of epicardial pacing, so the intrinsic conduction pathways were not physiologically engaged. This is a possible explanation for why we did not observe delayed conduction in the right ventricle, even though the right ventricular outflow tract is canonically associated with the site of arrhythmogenesis in patients with BrS. Importantly, it is not known whether the delayed conduction is due to inexcitability caused by extracellular epicardial stimulation, since it always occurred adjacent to the site of pacing, or if the response is functional conduction block. Subsequent study of the effects of pacing frequency on conduction slowing may assist in further characterizing the conduction block observed here.

Surprisingly, conduction did not respond similarly when hearts were challenged with mannitol in the context of hyponatremia. Although both mannitol and hyponatremia independently resulted in slowed CV for both genotypes (though to a greater extent for *Scn5a*^+/−^), the combination of the two interventions appears to attenuate this effect. Specifically, mannitol attenuated hyponatremia-induced conduction slowing independent of Na_v_1.5 functional expression.

Precisely identifying the mechanism by which mannitol ameliorates hyponatremic conduction slowing is beyond the scope of this study, but we can offer a few hypotheses. Notably, prior work in Langendorff-perfused guinea pig hearts demonstrates that ventricular conduction slowing during hyponatremia is due to reduced extracellular sodium per se, and not hypoosmolality, sodium calcium exchanger (NCX) activity, stretch-activated channels, or reduced chloride concentration (43). We previously published evidence that suggests mannitol-induced perinexal widening slows CV (35, 36, 38). However, reducing extracellular sodium decreases the equilibrium potential of sodium, which in turn may alter the biphasic relationship between CV and *W*_P_. Alternatively, reducing extracellular sodium can indirectly increase intracellular calcium by decreasing the forward rate of NCX (44). Increased intracellular calcium has repeatedly been shown to alter posttranslational modifications of a number of ion channels, which may in turn modify CV (45). In addition, a decrease in the forward rate of NCX may result in the reduction of extracellular calcium in diffusion limited nanodomains such as the perinexus. Given the dependence of extracellular adhesion on extracellular calcium concentrations, hyponatremia may indirectly modify *W*_P_, ultimately reducing CV to a range that is less sensitive to perinexal widening (17, 26). Finally, recent work suggests that perinexal widening may result in redistribution of associated ion channels. Specifically, edema-induced perinexal widening was associated with more diffuse Na_v_1.5 expression throughout the intercalated disc, relative to the dense expression near gap junctions seen in control tissue (46). Theoretically, a smaller population of Na_v_1.5 may provide relief for the source-sink mismatch of sodium ion availability that results from hyponatremia. Regardless of the specific hypothesis, the surprising finding that mannitol appears to rescue conduction delay in the *Scn5a*^+/−^ mouse heart requires additional investigation.

### Limitations

A number of limitations are inherent to the use of ex vivo Langendorff-perfused hearts. These include removal of autonomic inputs that may modulate determinants of conduction, as well as the nonworking preparation. In addition, the optical mapping experiments used blebbistatin as an electromechanical uncoupler. Like all electromechanical uncouplers, blebbistatin has been associated with off-target effects, such as action potential duration (APD) prolongation via altered intracellular calcium handling (47–49).

### Conclusion

To the best of our knowledge, this is the first preclinical evidence that hyponatremia and mannitol independently induce conduction deficits in a Na_v_1.5 LOF model. These data may suggest that exposure to either hyponatremia or mannitol could be detrimental to patients with Na_v_1.5 LOF. However, without a mechanistic understanding of how hyponatremia attenuates conduction slowing secondary to mannitol, caution should be exercised before attempting clinical translation. Nevertheless, these data add to a growing body of literature that suggests modulating ephaptic coupling may be a viable diagnostic or therapeutic intervention for diseases of reduced sodium channel function.

## DATA AVAILABILITY

Data will be made available upon reasonable request.

## SUPPLEMENTAL DATA

10.6084/m9.figshare.24944835 Supplemental Figs. S1 and S2: https://doi.org/10.6084/m9.figshare.24944835.

## GRANTS

This work was supported by National Heart, Lung, and Blood Institute Grants F31HL160172 (to G.A.B.), R01HL159097 (to S.P.), and R01HL102298 (to S.P.).

## DISCLOSURES

No conflicts of interest, financial or otherwise, are declared by the authors.

## AUTHOR CONTRIBUTIONS

G.A.B. and S.P. conceived and designed research; G.A.B., X.W., C.B., and M.W. performed experiments; G.A.B., X.W., and C.B. analyzed data; G.A.B., C.B., G.S.H., and S.P. interpreted results of experiments; G.A.B. and C.B. prepared figures; G.A.B. drafted manuscript; G.A.B., X.W., G.S.H., and S.P. edited and revised manuscript; G.A.B., X.W., C.B., M.W., G.S.H., and S.P. approved final version of manuscript.
